# Exostosis

**Published:** 1897-05

**Authors:** Douglas E. Caush


### SELECTIONS.

### Exostosis.

BY DOUGLAS E. CAUSH, L. D. S. I.

In introducing this subject of exostosis, it is our desire to draw your attention more especially to those changes that are observed in the examination of teeth by the aid of the microscope; and, to make ourselves clear, it may be as well, at this early period of our paper, to state the method adopted in preparing the teeth for examination. If the tooth to be examined has been out of the mouth for some time, it is at once cut into sections by the aid of a fret saw, commencing at the apex of the root: each section being made as thin as possible, and as it is cut off, placed in a watch glass containing water (each watch glass is numbered so that the section retains its individual number until mounted). The method employed in preparing the sections is that suggested by Mr. T. Charters White.

If, on the other hand, the tooth has been recently extracted, we place it into an alcoholic solution of rubin, and allow it to remain for some weeks, so that the colored solution may penetrate into the tubuli, and any other spaces that are to be found in the tooth; it is then cut and prepared in the same manner as the dry sections, and by this method very beautiful, as well as very perfect, sections, showing tubuli, &c., may be obtained.

After the sections are thin enough for our purpose of examination, we stain them by what we call the surface method. It is done as follows. Into a watch glass, containing an aqueous solution of rubin, the section is placed for about two minutes, it is then taken out, and the surface moisture dry from the section, which is then mounted in hardened Canada balsam. By adopting this method, the section becomes stained in the following manner: all soft tissue wherever found is stained a deep red color; the cementum is stained red; the dentine does not take up the stain at all; and thus we have a perfect differentiation of the tissues. Having described the method of preparing our slides, we will now direct your attention to some of the changes that can be observed. To better understand the changes it is well to keep clearly in mind the normal condition of the tissues. The first change that takes place in the development of exostosis is that of the thickening of the alveolar-dental membrane. This is usually caused by some slight irritation; it may be from a filling, or alteration of the articulation, produced either by decay or the loss of other teeth, or from some similar cause. In all cases the result is the same, namely, an increase in the blood supply to the part; this, in its turn, causes increased activity in the cells of the membrane.

Should we at any time have the opportunity of microscopically examining such a tooth, we shall find the membrane much more colored than usual, as well as increased in thickness, owing to the cells in the portion of the membrane attached to the cementum having become more and more active. These cells continuously break up into new cells, and these new cells divide and sub-divide until the membrane has a much larger number of cells than is found in its normal condition; the natural result being that a certain amount of pressure is brought to bear upon the cementum at this point. As this pressure increases from the development of the new cells, many of the cells change their character from that of an ordinary mucleated cell to that of a cementoclast, or giant cell, having the power of absorbing the cementum at the point of contact. If the blood supply becomes normal at this stage no further change takes place; but should the irritation be kept up, the giant cells continue their activity. Should the condition become chronic, the giant cells thus formed will continue to absorb the cementum, until the whole of it is absorbed away, after which the giant cells pass into the granular layer of the dentine, and there continue their work, until a portion, or the whole of this layer, is absorbed. This absorption takes place in an irregular manner, owing to the difference in the density of the tissue, this difference of density being caused by the more or less perfect calcification of the dentine; on the osteoclasts having penetrated to the denser portion of the tissue the absorption ceases, and the cells themselves again change their character from cementoclasts to cementoblasts, or cementum-depositing cells, and begin their functions of depositing cementum into the spaces previously produced by the absorption. It is not at all an unusual thing to find a number of these cells retaining their soft structure, surrounded by cementum, as if some of the cementoclasts had been slow to change their character, thus leaving portions of soft tissue in the newly-deposited cementum. The new tissue is usually deposited in layers, differing from the original cemental tissue in that it contains a large number of lacunae and canaliculi, these frequently occupying the position previously occupied by the original layer of cementum. This layer of cementum continues to be deposited as long as the blood brings to the cells material for the development of the tissue. The amount deposited varies much; it may be but a very thin layer, or it may be continuously deposited, until it is many times as thick as the original layer of cementum.

That the development of this tissue is not always continuous may be proved by the manifestation of a number of lines in the section; in these lines we find there are few or no lacunae, and where the calcification of the new tissue is evidently much more perfect; thus showing that more perfect calcification has evidently taken place during the time of rest from development, this time of rest being caused by the cessation of the irritation to the alveolar-dental membrane, and, as a consequence, slow, but more perfect calcification of the cementum; where this has taken place, it usually leaves a structureless layer of tissue. When these lines exist they are as a rule most irregularly placed, varying very much in their distance one from another, hence the more continuous the deposition the fewer the lines. It is an unusual thing to find in some sections, after these periods of rest, that a portion of the new tissue has been absorbed away, leaving semilunar markings, which are filled later on with new tissue.

When the development of this tissue is rapid we usually find a number of large lacunae with their canaliculi anastomosing with the canaliculi of the surrounding lacunae. If, on the other hand, the deposition of this tissue has been slow, the lucanae are much smaller and fewer in number, with, as a rule, smaller canaliculi; under such circumstances the lacunae may be isolated and surrounded by tissue, structureless in character; or should the deposition of any one portion of the tissue be more slow than that of the surrounding tissue, the same thing occurs, we get not only more perfect calcification at that point, but an irregular outline of the tissue is produced. It is not at all an unusual thing to find a number of large lacunae formed immediately after one of these marks produced by the time of rest, the lacunae gradually becoming less in number until there is another period of rest, so that on examining the section we are able to define the way in which the new tissue has been deposited. These changes may continue for months or even years, and leave behind them indelible markings in the tooth thus affected.

Instead of all, or any of these changes taking place, the point of absorption may be very much restricted in area, or the aborption may commence at that part of the root where the calcification of the original tissue has been imperfect, and as a result of this we have a deep yet restricted area of absorption taken place. This goes on until it reaches the granular layer of the dentine, and here, frequently at that point where the absorption has reached the dentine, is a portion of softened or uncalcified tissue; this rapidly dissolved away by the cementoclasts, and thus a deeper and somewhat semi-lunar-shaped cavity is produced. As soon as the dense tissue opposes the cementoclasts they cease their functions, and after a time a new tissue, cemental in character, is produced, until the whole of the space is filled with cementum.

The great difference between this and the absorption, as seen in the earlier stages of exostosis, is the definite line of demarcation produced by the absorption, both in the cementum and dentine, as contrasted with the irregular line of absorption seem in general exostosis. Mr. George Henry of Hastings, was, I believe, the first to draw attention to this alteration of the tissues, and gave to it the name of inostosis.

So far as our microscopic slides show, this form of absorption takes place nearer the neck of the tooth than exostosis usually commences; and, we think, this may be one explanation of the cause of this restricted area of absorption, all the surrounding tissue being more dense near the neck than at or near the apex of the root; it is, therefore, only when the inflammation of the alveolar-dental membrane occurs over an imperfectly calcified portion of the tooth that this absorption takes place. We have in our collection a number of slides showing that this form of absorption sometimes takes place after there has been a certain amount of general exostosis, the absorption passing through the deposited cemental tissue into the dentine, and in this space another layer of cementum is deposited. In some cases we have no doubt that this absorption is the result of an unusually severe attack of periostitis. At the same time we must be careful not to confound these markings with those produced by the more acute form of inflammation and suppuration known as alveolar abscess.

Wherever there is a chronic abscess near or pressing upon the roots of the tooth, we have absorption, and this absorption is, as a rule, more general in character, and covers a larger area than in those cases known as inostosis. In these cases it is not at all unusual to find, not only the cementum absorbed away, but the absorption may have gone into the dentine, and in some extreme cases we have found the absorption has not ceased until the pulp canal has not only been reached, but even a portion of that has been absorbed away. Again, in some of these cases, nature appears to have tried to produce a remedy by depositing in these depressions new tissue, cemental in character.

We also find in many cases the canaliculi of the lacunae anastomising with some of the finer branches of the tubuli, and thus forming a network of minute canals from the cementum to the pulp canal.

We have also found in many cases where there has been a large deposition of new tissue, that this tissue has enclosed within it a number of canals, passing in different directions; these canals usually have a lining membrane, and to all appearances are blood vessels of the alveolus and membrane.

There is also to be found at times canals of quite another character, passing directly from the pulp canal, at right or acute angles to the canal, through the dentine, into the cementum. In this case the contents of the canals are similar in character to the pulp itself; in some cases we have seen these canals enlarged, and a layer of cementum of varying thickness lining the canal.

These last canals lead us to the pulp, and we wish now for a short time to draw your attention to certain changes we have found in the dentine forming the pulp canal. It is just possible that these changes take place in teeth that are not exostosed, but as our examination has been principally of exostosed teeth, we should be glad if others could give any information as to whether similar changes have taken place in the teeth that are not exostosed.

If we examine a tooth with an inflamed pulp, we shall probably find that the following changes have taken place in the pulp:— In the layer of odontoblast cells that form the connecting link between the pulp proper and the dentine, we find, at that point where the inflammation is most acute, the individual odontoblast cell has been altered in character and appearance; there is an absence of the usual processes, especially those which pass into the dentine, and instead of these we have a cell somewhat oval in shape, and frequently the cells are increased in number, and apparently the connection between the dentine and these cells have ceased. This change may be restricted in area; if the inflammation of the pulp has been either very acute or chronic, the cells themselves increase rapidly by cell division, and a certain amount of pressure is produced upon the dentine. The result of the cutting off of the supply of nourishment from the restricted area is to produce those transparent zones so frequently seen in microscopic sections of teeth where there are apparently no tubuli radiating from that portion of the pulp canal in which these changes have taken place. This may spread until the whole of the dentine between the pulp canal and the cementum in this restricted area is apparently structureless. We believe that this change is brought about by the more perfect calcification of this portion of the dentine. Should, however, the pulp continue inflamed, we find the original odontoblastic layer becoming thickened, and after a time, a number of giant cells are developed in the thickened layer; these cells pressing upon the dentine cause a certain amount of absorption of the latter, and this absorption may be restricted to a single point, or it may spread laterally so that two or three canals in the roots of molar teeth may be united into one irregular canal. There appears to be no restriction as to the way in which the absorption will spread from the canals, so varied is it that almost every section shows an alteration in the conformation of the canal.

After a time another change takes place in the character of the cells forming the outer layer of the inflamed pulp; and, instead of osteoclasts, we have cells capable of depositing a tissue containing lacunae and canaliculi exactly in appearance like the *cemental* tissue found on the outside of the dentine; this tissue is quite distinct in character from the surrounding dentine, and the line of demarcation between the two is usually very pronounced. This new tissue may vary very much in quantity from that of a very thin layer to a mass large enough to almost fill the pulp canal and the spaces excavated. That this tissue is not secondary dentine, we think, will be clearly demonstrated by comparing the slides that have just passed before you and the next two, which show secondary dentine in both transverse and longitudinal sections.

You will notice in the former slides the new tissue fills up *semi-lunar* spaces where the cut ends of the tubuli of the dentine end abruptly; in the secondary dentine there are no *semi-lunar* markings, and the tubuli of the secondary tissue are more or less connected with the tubuli of the original dentine; in the secondary dentine the tissue is always added *directly* to the old tissue without any previous absorption, and, as a consequence gradually from the commencement, of the deposit, decreases the size of the canal; this is reversed in the case of the new tissue, as the deposition is always preceded by absorption, and usually contains lacunae and canaliculi with *no* tubuli.

That exostosis is not of a recent date, I have had the opportunity of proving lately, thanks to the kindness of Charles Dawson, Esq., F. G. S., F. S. A., he having placed at my disposal, for the purpose of making a microscopic examination, two molar teeth, found by him in the chalk *debris* within the artificially excavated caverns at Hayes Down, Lavant. With the teeth were associated some flint implements of the later Neolithic type; the age of the teeth probably is *not later* than 2,000 years, and perhaps much earlier. In both of them we found exostosis, and in one of them certainly excavations both on the outside of the cementum and in the pulp canal. We have also found that these, or similar changes, have taken *place* in the horse, with even a greater intensity than in those of the human race.

